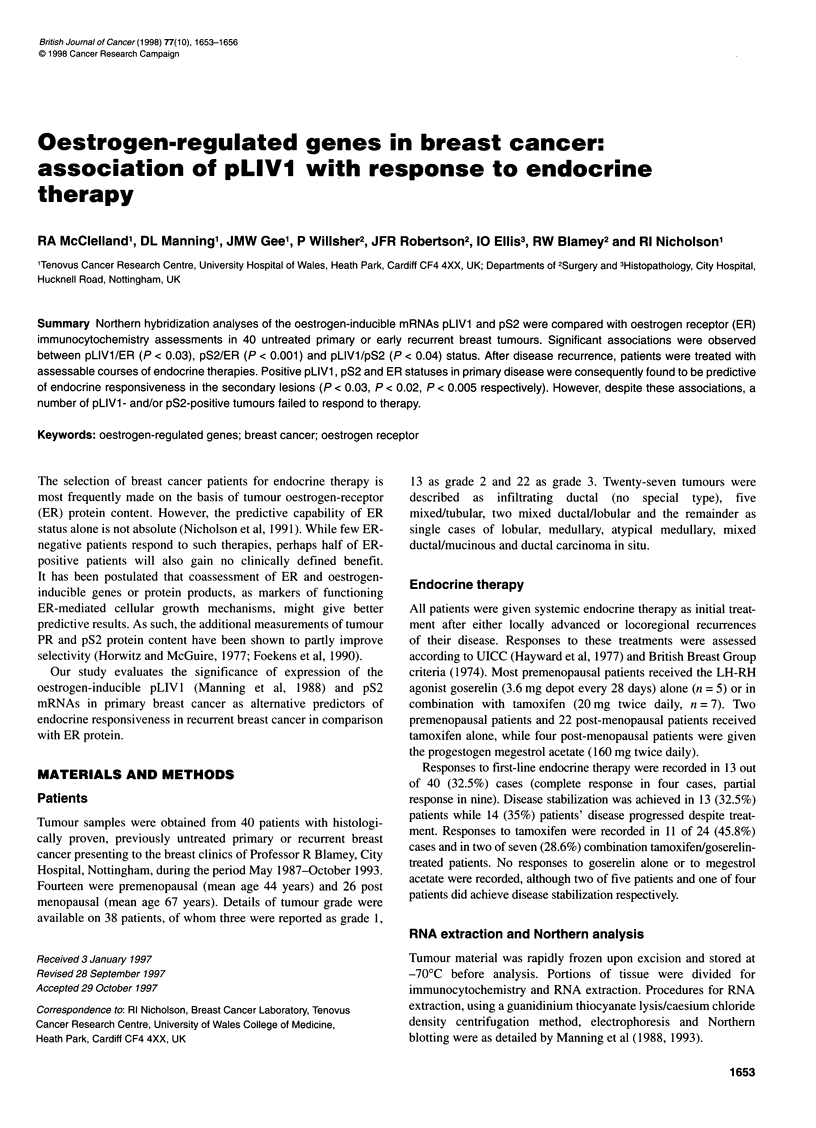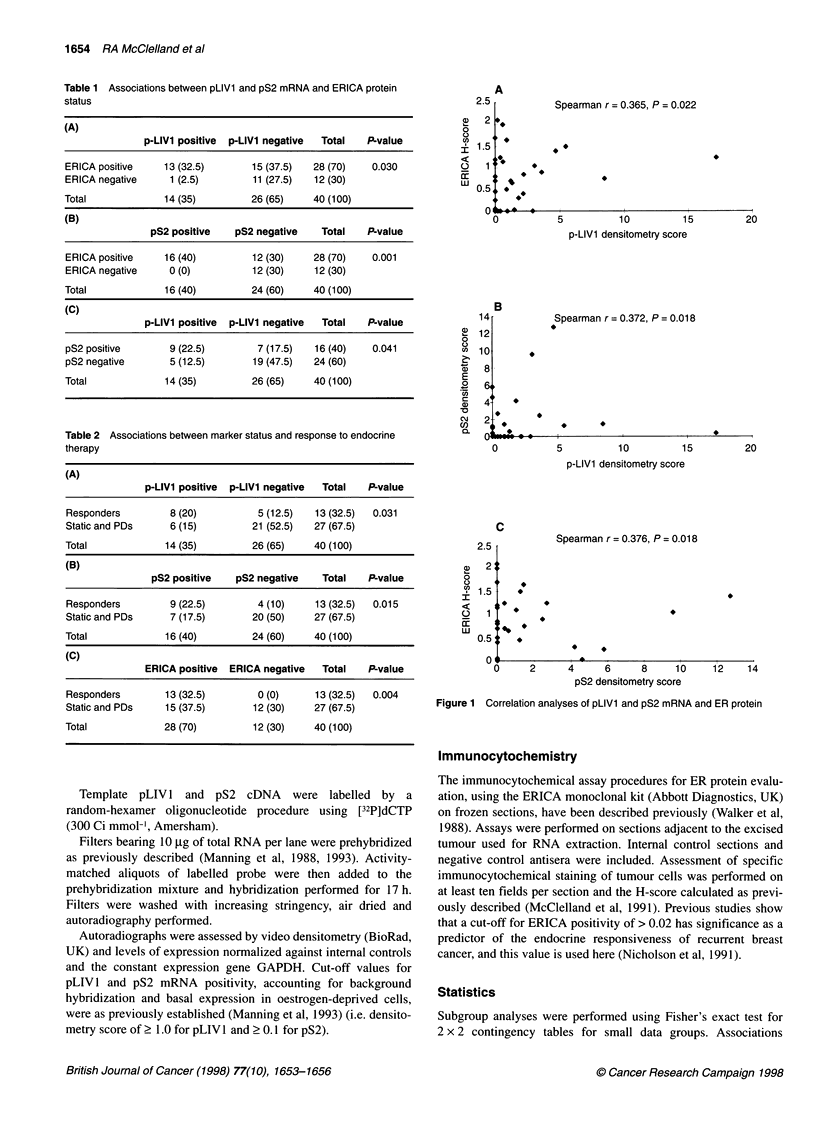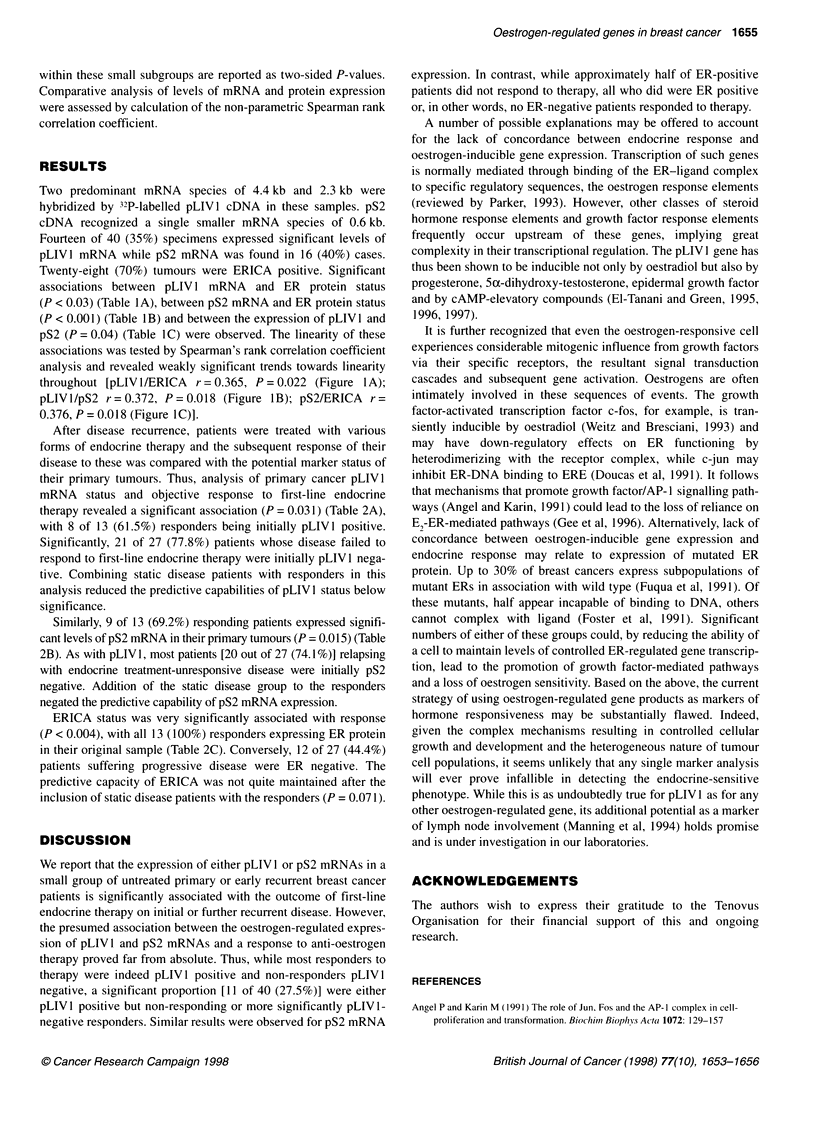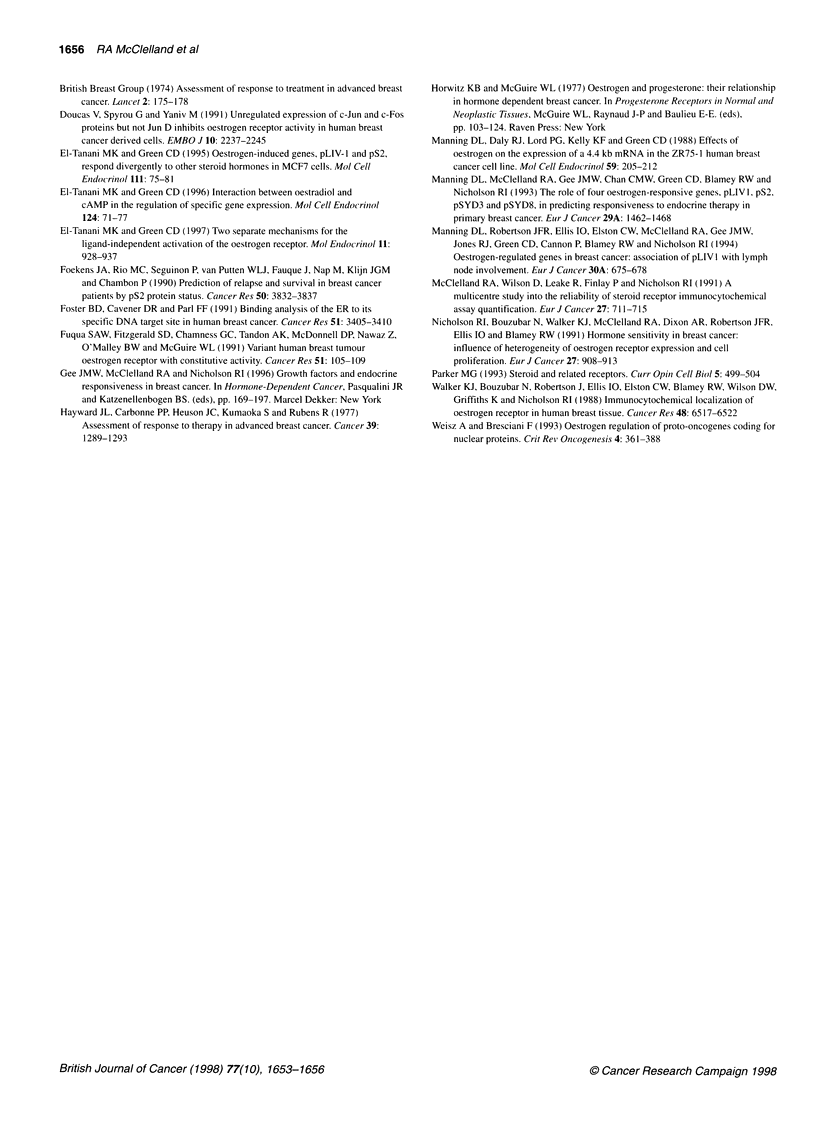# Oestrogen-regulated genes in breast cancer: association of pLIV1 with response to endocrine therapy.

**DOI:** 10.1038/bjc.1998.271

**Published:** 1998-05

**Authors:** R. A. McClelland, D. L. Manning, J. M. Gee, P. Willsher, J. F. Robertson, I. O. Ellis, R. W. Blamey, R. I. Nicholson

**Affiliations:** Tenovus Cancer Research Centre, University Hospital of Wales, Heath Park, Cardiff, UK.

## Abstract

Northern hybridization analyses of the oestrogen-inducible mRNAs pLIV1 and pS2 were compared with oestrogen receptor (ER) immunocytochemistry assessments in 40 untreated primary or early recurrent breast tumours. Significant associations were observed between pLIV1/ER (P < 0.03), pS2/ER (P < 0.001) and pLIV1/pS2 (P < 0.04) status. After disease recurrence, patients were treated with assessable courses of endocrine therapies. Positive pLIV1, pS2 and ER statuses in primary disease were consequently found to be predictive of endocrine responsiveness in the secondary lesions (P < 0.03, P < 0.02, P < 0.005 respectively). However, despite these associations, a number of pLIV1- and/or pS2-positive tumours failed to respond to therapy.


British Journal of Cancer (1998) 77(10), 1653–1656
© 1998 Cancer Research Campaign

### Oestrogen-regulated genes in breast cancer: association of pLIV1 with response to endocrine therapy

**RA McClelland[1], DL Manning[1], JMW Gee[1], P Willsher[2], JFR Robertson[2], IO Ellis[3], RW Blamey[2] and RI Nicholson[1]**

[1]Tenovus Cancer Research Centre, University Hospital of Wales, Heath Park, Cardiff CF4 4XX, UK; Departments of [2]Surgery and [3]Histopathology, City Hospital, Hucknell Road, Nottingham, UK

**Summary** Northern hybridization analyses of the oestrogen-inducible mRNAs pLIV1 and pS2 were compared with oestrogen receptor (ER) immunocytochemistry assessments in 40 untreated primary or early recurrent breast tumours. Significant associations were observed between pLIV1/ER ($P < 0.03$), pS2/ER ($P < 0.001$) and pLIV1/pS2 ($P < 0.04$) status. After disease recurrence, patients were treated with assessable courses of endocrine therapies. Positive pLIV1, pS2 and ER statuses in primary disease were consequently found to be predictive of endocrine responsiveness in the secondary lesions ($P < 0.03$, $P < 0.02$, $P < 0.005$ respectively). However, despite these associations, a number of pLIV1- and/or pS2-positive tumours failed to respond to therapy.

**Keywords:** oestrogen-regulated genes; breast cancer; oestrogen receptor

The selection of breast cancer patients for endocrine therapy is most frequently made on the basis of tumour oestrogen-receptor (ER) protein content. However, the predictive capability of ER status alone is not absolute (Nicholson et al, 1991). While few ER-negative patients respond to such therapies, perhaps half of ER-positive patients will also gain no clinically defined benefit. It has been postulated that coassessment of ER and oestrogen-inducible genes or protein products, as markers of functioning ER-mediated cellular growth mechanisms, might give better predictive results. As such, the additional measurements of tumour PR and pS2 protein content have been shown to partly improve selectivity (Horwitz and McGuire, 1977; Foekens et al, 1990).

Our study evaluates the significance of expression of the oestrogen-inducible pLIV1 (Manning et al, 1988) and pS2 mRNAs in primary breast cancer as alternative predictors of endocrine responsiveness in recurrent breast cancer in comparison with ER protein.

### MATERIALS AND METHODS

### Patients

Tumour samples were obtained from 40 patients with histologically proven, previously untreated primary or recurrent breast cancer presenting to the breast clinics of Professor R Blamey, City Hospital, Nottingham, during the period May 1987–October 1993. Fourteen were premenopausal (mean age 44 years) and 26 post menopausal (mean age 67 years). Details of tumour grade were available on 38 patients, of whom three were reported as grade 1, 13 as grade 2 and 22 as grade 3. Twenty-seven tumours were described as infiltrating ductal (no special type), five mixed/tubular, two mixed ductal/lobular and the remainder as single cases of lobular, medullary, atypical medullary, mixed ductal/mucinous and ductal carcinoma in situ.

*Received 3 January 1997*
*Revised 28 September 1997*
*Accepted 29 October 1997*

*Correspondence to*: RI Nicholson, Breast Cancer Laboratory, Tenovus Cancer Research Centre, University of Wales College of Medicine, Heath Park, Cardiff CF4 4XX, UK

### Endocrine therapy

All patients were given systemic endocrine therapy as initial treatment after either locally advanced or locoregional recurrences of their disease. Responses to these treatments were assessed according to UICC (Hayward et al, 1977) and British Breast Group criteria (1974). Most premenopausal patients received the LH-RH agonist goserelin (3.6 mg depot every 28 days) alone ($n = 5$) or in combination with tamoxifen (20 mg twice daily, $n = 7$). Two premenopausal patients and 22 post-menopausal patients received tamoxifen alone, while four post-menopausal patients were given the progestogen megestrol acetate (160 mg twice daily).

Responses to first-line endocrine therapy were recorded in 13 out of 40 (32.5%) cases (complete response in four cases, partial response in nine). Disease stabilization was achieved in 13 (32.5%) patients while 14 (35%) patients' disease progressed despite treatment. Responses to tamoxifen were recorded in 11 of 24 (45.8%) cases and in two of seven (28.6%) combination tamoxifen/goserelin-treated patients. No responses to goserelin alone or to megestrol acetate were recorded, although two of five patients and one of four patients did achieve disease stabilization respectively.

### RNA extraction and Northern analysis

Tumour material was rapidly frozen upon excision and stored at −70°C before analysis. Portions of tissue were divided for immunocytochemistry and RNA extraction. Procedures for RNA extraction, using a guanidinium thiocyanate lysis/caesium chloride density centrifugation method, electrophoresis and Northern blotting were as detailed by Manning et al (1988, 1993).

**Table 1** Associations between pLIV1 and pS2 mRNA and ERICA protein status

| (A) | p-LIV1 positive | p-LIV1 negative | Total | *P*-value |
|---|---|---|---|---|
| ERICA positive | 13 (32.5) | 15 (37.5) | 28 (70) | 0.030 |
| ERICA negative | 1 (2.5) | 11 (27.5) | 12 (30) | |
| Total | 14 (35) | 26 (65) | 40 (100) | |

| (B) | pS2 positive | pS2 negative | Total | *P*-value |
|---|---|---|---|---|
| ERICA positive | 16 (40) | 12 (30) | 28 (70) | 0.001 |
| ERICA negative | 0 (0) | 12 (30) | 12 (30) | |
| Total | 16 (40) | 24 (60) | 40 (100) | |

| (C) | p-LIV1 positive | p-LIV1 negative | Total | *P*-value |
|---|---|---|---|---|
| pS2 positive | 9 (22.5) | 7 (17.5) | 16 (40) | 0.041 |
| pS2 negative | 5 (12.5) | 19 (47.5) | 24 (60) | |
| Total | 14 (35) | 26 (65) | 40 (100) | |

**Table 2** Associations between marker status and response to endocrine therapy

| (A) | p-LIV1 positive | p-LIV1 negative | Total | *P*-value |
|---|---|---|---|---|
| Responders | 8 (20) | 5 (12.5) | 13 (32.5) | 0.031 |
| Static and PDs | 6 (15) | 21 (52.5) | 27 (67.5) | |
| Total | 14 (35) | 26 (65) | 40 (100) | |

| (B) | pS2 positive | pS2 negative | Total | *P*-value |
|---|---|---|---|---|
| Responders | 9 (22.5) | 4 (10) | 13 (32.5) | 0.015 |
| Static and PDs | 7 (17.5) | 20 (50) | 27 (67.5) | |
| Total | 16 (40) | 24 (60) | 40 (100) | |

| (C) | ERICA positive | ERICA negative | Total | *P*-value |
|---|---|---|---|---|
| Responders | 13 (32.5) | 0 (0) | 13 (32.5) | 0.004 |
| Static and PDs | 15 (37.5) | 12 (30) | 27 (67.5) | |
| Total | 28 (70) | 12 (30) | 40 (100) | |

**Figure 1** Correlation analyses of pLIV1 and pS2 mRNA and ER protein

Template pLIV1 and pS2 cDNA were labelled by a random-hexamer oligonucleotide procedure using [$^{32}$P]dCTP (300 Ci mmol$^{-1}$, Amersham).

Filters bearing 10 µg of total RNA per lane were prehybridized as previously described (Manning et al, 1988, 1993). Activity-matched aliquots of labelled probe were then added to the prehybridization mixture and hybridization performed for 17 h. Filters were washed with increasing stringency, air dried and autoradiography performed.

Autoradiographs were assessed by video densitometry (BioRad, UK) and levels of expression normalized against internal controls and the constant expression gene GAPDH. Cut-off values for pLIV1 and pS2 mRNA positivity, accounting for background hybridization and basal expression in oestrogen-deprived cells, were as previously established (Manning et al, 1993) (i.e. densitometry score of $\geq$ 1.0 for pLIV1 and $\geq$ 0.1 for pS2).

### Immunocytochemistry

The immunocytochemical assay procedures for ER protein evaluation, using the ERICA monoclonal kit (Abbott Diagnostics, UK) on frozen sections, have been described previously (Walker et al, 1988). Assays were performed on sections adjacent to the excised tumour used for RNA extraction. Internal control sections and negative control antisera were included. Assessment of specific immunocytochemical staining of tumour cells was performed on at least ten fields per section and the H-score calculated as previously described (McClelland et al, 1991). Previous studies show that a cut-off for ERICA positivity of > 0.02 has significance as a predictor of the endocrine responsiveness of recurrent breast cancer, and this value is used here (Nicholson et al, 1991).

### Statistics

Subgroup analyses were performed using Fisher's exact test for 2 × 2 contingency tables for small data groups. Associations

© Cancer Research Campaign 1998

within these small subgroups are reported as two-sided *P*-values. Comparative analysis of levels of mRNA and protein expression were assessed by calculation of the non-parametric Spearman rank correlation coefficient.

### RESULTS

Two predominant mRNA species of 4.4 kb and 2.3 kb were hybridized by $^{32}$P-labelled pLIV1 cDNA in these samples. pS2 cDNA recognized a single smaller mRNA species of 0.6 kb. Fourteen of 40 (35%) specimens expressed significant levels of pLIV1 mRNA while pS2 mRNA was found in 16 (40%) cases. Twenty-eight (70%) tumours were ERICA positive. Significant associations between pLIV1 mRNA and ER protein status ($P < 0.03$) (Table 1A), between pS2 mRNA and ER protein status ($P < 0.001$) (Table 1B) and between the expression of pLIV1 and pS2 ($P = 0.04$) (Table 1C) were observed. The linearity of these associations was tested by Spearman's rank correlation coefficient analysis and revealed weakly significant trends towards linearity throughout [pLIV1/ERICA $r = 0.365$, $P = 0.022$ (Figure 1A); pLIV1/pS2 $r = 0.372$, $P = 0.018$ (Figure 1B); pS2/ERICA $r = 0.376$, $P = 0.018$ (Figure 1C)].

After disease recurrence, patients were treated with various forms of endocrine therapy and the subsequent response of their disease to these was compared with the potential marker status of their primary tumours. Thus, analysis of primary cancer pLIV1 mRNA status and objective response to first-line endocrine therapy revealed a significant association ($P = 0.031$) (Table 2A), with 8 of 13 (61.5%) responders being initially pLIV1 positive. Significantly, 21 of 27 (77.8%) patients whose disease failed to respond to first-line endocrine therapy were initially pLIV1 negative. Combining static disease patients with responders in this analysis reduced the predictive capabilities of pLIV1 status below significance.

Similarly, 9 of 13 (69.2%) responding patients expressed significant levels of pS2 mRNA in their primary tumours ($P = 0.015$) (Table 2B). As with pLIV1, most patients [20 out of 27 (74.1%)] relapsing with endocrine treatment-unresponsive disease were initially pS2 negative. Addition of the static disease group to the responders negated the predictive capability of pS2 mRNA expression.

ERICA status was very significantly associated with response ($P < 0.004$), with all 13 (100%) responders expressing ER protein in their original sample (Table 2C). Conversely, 12 of 27 (44.4%) patients suffering progressive disease were ER negative. The predictive capacity of ERICA was not quite maintained after the inclusion of static disease patients with the responders ($P = 0.071$).

### DISCUSSION

We report that the expression of either pLIV1 or pS2 mRNAs in a small group of untreated primary or early recurrent breast cancer patients is significantly associated with the outcome of first-line endocrine therapy on initial or further recurrent disease. However, the presumed association between the oestrogen-regulated expression of pLIV1 and pS2 mRNAs and a response to anti-oestrogen therapy proved far from absolute. Thus, while most responders to therapy were indeed pLIV1 positive and non-responders pLIV1 negative, a significant proportion [11 of 40 (27.5%)] were either pLIV1 positive but non-responding or more significantly pLIV1-negative responders. Similar results were observed for pS2 mRNA expression. In contrast, while approximately half of ER-positive patients did not respond to therapy, all who did were ER positive or, in other words, no ER-negative patients responded to therapy.

A number of possible explanations may be offered to account for the lack of concordance between endocrine response and oestrogen-inducible gene expression. Transcription of such genes is normally mediated through binding of the ER–ligand complex to specific regulatory sequences, the oestrogen response elements (reviewed by Parker, 1993). However, other classes of steroid hormone response elements and growth factor response elements frequently occur upstream of these genes, implying great complexity in their transcriptional regulation. The pLIV1 gene has thus been shown to be inducible not only by oestradiol but also by progesterone, 5α-dihydroxy-testosterone, epidermal growth factor and by cAMP-elevatory compounds (El-Tanani and Green, 1995, 1996, 1997).

It is further recognized that even the oestrogen-responsive cell experiences considerable mitogenic influence from growth factors via their specific receptors, the resultant signal transduction cascades and subsequent gene activation. Oestrogens are often intimately involved in these sequences of events. The growth factor-activated transcription factor c-fos, for example, is transiently inducible by oestradiol (Weitz and Bresciani, 1993) and may have down-regulatory effects on ER functioning by heterodimerizing with the receptor complex, while c-jun may inhibit ER-DNA binding to ERE (Doucas et al, 1991). It follows that mechanisms that promote growth factor/AP-1 signalling pathways (Angel and Karin, 1991) could lead to the loss of reliance on $E_2$-ER-mediated pathways (Gee et al, 1996). Alternatively, lack of concordance between oestrogen-inducible gene expression and endocrine response may relate to expression of mutated ER protein. Up to 30% of breast cancers express subpopulations of mutant ERs in association with wild type (Fuqua et al, 1991). Of these mutants, half appear incapable of binding to DNA, others cannot complex with ligand (Foster et al, 1991). Significant numbers of either of these groups could, by reducing the ability of a cell to maintain levels of controlled ER-regulated gene transcription, lead to the promotion of growth factor-mediated pathways and a loss of oestrogen sensitivity. Based on the above, the current strategy of using oestrogen-regulated gene products as markers of hormone responsiveness may be substantially flawed. Indeed, given the complex mechanisms resulting in controlled cellular growth and development and the heterogeneous nature of tumour cell populations, it seems unlikely that any single marker analysis will ever prove infallible in detecting the endocrine-sensitive phenotype. While this is as undoubtedly true for pLIV1 as for any other oestrogen-regulated gene, its additional potential as a marker of lymph node involvement (Manning et al, 1994) holds promise and is under investigation in our laboratories.

### ACKNOWLEDGEMENTS

The authors wish to express their gratitude to the Tenovus Organisation for their financial support of this and ongoing research.

### REFERENCES

Angel P and Karin M (1991) The role of Jun, Fos and the AP-1 complex in cell-proliferation and transformation. *Biochim Biophys Acta* **1072**: 129–157

© Cancer Research Campaign 1998

British Breast Group (1974) Assessment of response to treatment in advanced breast cancer. *Lancet* **2**: 175–178

Doucas V, Spyrou G and Yaniv M (1991) Unregulated expression of c-Jun and c-Fos proteins but not Jun D inhibits oestrogen receptor activity in human breast cancer derived cells. *EMBO J* **10**: 2237–2245

El-Tanani MK and Green CD (1995) Oestrogen-induced genes, pLIV-1 and pS2, respond divergently to other steroid hormones in MCF7 cells. *Mol Cell Endocrinol* **111**: 75–81

El-Tanani MK and Green CD (1996) Interaction between oestradiol and cAMP in the regulation of specific gene expression. *Mol Cell Endocrinol* **124**: 71–77

El-Tanani MK and Green CD (1997) Two separate mechanisms for the ligand-independent activation of the oestrogen receptor. *Mol Endocrinol* **11**: 928–937

Foekens JA, Rio MC, Seguinon P, van Putten WLJ, Fauque J, Nap M, Klijn JGM and Chambon P (1990) Prediction of relapse and survival in breast cancer patients by pS2 protein status. *Cancer Res* **50**: 3832–3837

Foster BD, Cavener DR and Parl FF (1991) Binding analysis of the ER to its specific DNA target site in human breast cancer. *Cancer Res* **51**: 3405–3410

Fuqua SAW, Fitzgerald SD, Chamness GC, Tandon AK, McDonnell DP, Nawaz Z, O'Malley BW and McGuire WL (1991) Variant human breast tumour oestrogen receptor with constitutive activity. *Cancer Res* **51**: 105–109

Gee JMW, McClelland RA and Nicholson RI (1996) Growth factors and endocrine responsiveness in breast cancer. In *Hormone-Dependent Cancer*, Pasqualini JR and Katzenellenbogen BS. (eds), pp. 169–197. Marcel Dekker: New York

Hayward JL, Carbonne PP, Heuson JC, Kumaoka S and Rubens R (1977) Assessment of response to therapy in advanced breast cancer. *Cancer* **39**: 1289–1293

Horwitz KB and McGuire WL (1977) Oestrogen and progesterone: their relationship in hormone dependent breast cancer. In *Progesterone Receptors in Normal and Neoplastic Tissues*, McGuire WL, Raynaud J-P and Baulieu E-E. (eds), pp. 103–124. Raven Press: New York

Manning DL, Daly RJ, Lord PG, Kelly KF and Green CD (1988) Effects of oestrogen on the expression of a 4.4 kb mRNA in the ZR75-1 human breast cancer cell line. *Mol Cell Endocrinol* **59**: 205–212

Manning DL, McClelland RA, Gee JMW, Chan CMW, Green CD, Blamey RW and Nicholson RI (1993) The role of four oestrogen-responsive genes, pLIV1, pS2, pSYD3 and pSYD8, in predicting responsiveness to endocrine therapy in primary breast cancer. *Eur J Cancer* **29A**: 1462–1468

Manning DL, Robertson JFR, Ellis IO, Elston CW, McClelland RA, Gee JMW, Jones RJ, Green CD, Cannon P, Blamey RW and Nicholson RI (1994) Oestrogen-regulated genes in breast cancer: association of pLIV1 with lymph node involvement. *Eur J Cancer* **30A**: 675–678

McClelland RA, Wilson D, Leake R, Finlay P and Nicholson RI (1991) A multicentre study into the reliability of steroid receptor immunocytochemical assay quantification. *Eur J Cancer* **27**: 711–715

Nicholson RI, Bouzubar N, Walker KJ, McClelland RA, Dixon AR, Robertson JFR, Ellis IO and Blamey RW (1991) Hormone sensitivity in breast cancer: influence of heterogeneity of oestrogen receptor expression and cell proliferation. *Eur J Cancer* **27**: 908–913

Parker MG (1993) Steroid and related receptors. *Curr Opin Cell Biol* **5**: 499–504

Walker KJ, Bouzubar N, Robertson J, Ellis IO, Elston CW, Blamey RW, Wilson DW, Griffiths K and Nicholson RI (1988) Immunocytochemical localization of oestrogen receptor in human breast tissue. *Cancer Res* **48**: 6517–6522

Weisz A and Bresciani F (1993) Oestrogen regulation of proto-oncogenes coding for nuclear proteins. *Crit Rev Oncogenesis* **4**: 361–388

*© Cancer Research Campaign 1998*